# Is translational research compatible with preclinical publication strategies?

**DOI:** 10.1186/1748-717X-1-4

**Published:** 2006-03-24

**Authors:** Stig Linder, Maria C Shoshan

**Affiliations:** 1 Cancer Center Karolinska, Department of Oncology and Pathology, Karolinska Institute and Hospital, S-171 76 Stockholm, Sweden

## Abstract

The term "translational research" is used to describe the transfer of basic biological knowledge into practical medicine, a process necessary for motivation of public spending. In the area of cancer therapeutics, it is becoming increasingly evident that results obtained *in vitro *and in animal models are difficult to translate into clinical medicine. We here argue that a number of factors contribute to making the translation process inefficient. These factors include the use of sensitive cell lines and fast growing experimental tumors as targets for novel therapies, and the use of unrealistic drug concentrations and radiation doses. We also argue that aggressive interpretation of data, successful in hypothesis-building biological research, does not form a solid base for development of clinically useful treatment modalities. We question whether "clean" results obtained in simplified models, expected for publication in high-impact journals, represent solid foundations for improved treatment of patients. Open-access journals such as Radiation Oncology have a large mission to fulfill by publishing relevant data to be used for making actual progress in translational cancer research.

## Background

In a survey of clinical trials of potential anticancer drugs performed by Nygren and Larsson in 2003 [1], it was concluded that "in earlier phase (trials) no or modest anticancer activity was reported" and it was speculated that "the expanding knowledge in tumour biology might not easily translate into new substantially better anticancer drugs". This statement leads to questions of whether the process of translational research is slower than anticipated, and – if so – why this might be. One obvious factor is the complexity of biology; we do not yet quite understand all details with regard to how cancer cells work. How can we then expect to cure cancer? However, we here argue that translational cancer research might suffer from shortcomings, in academic laboratories in particular. We discuss a number of factors which we believe contribute. Our article is meant to be provocative.

## Mice are not men

The French Nobel laureate Jacques Monod remarked in 1965 that "What is true for *E. coli *is true for an elephant, only more so." One of the main outcomes of the genomic sequencing projects is the recognition that many genes, including those associated with various diseases in humans, are evolutionary conserved from yeast to man. Genomic sequence comparisons have revealed that 61% of *Drosophila melanogaster *and up to 97% mouse genes are similar to human genes. Many of the mechanisms developed by prokaryotic and eukaryotic cells to use energy, regulate gene expression and respond to environmental challenges utilize similar basic biochemical processes.

However, and significantly, there are important differences between mouse and human cells. Biological mechanisms that control life span (replicative senescence) and apoptosis are not perfectly conserved. It is well known that mouse cells easily become immortalized in culture, whereas human cells do not. More recent studies have shown that p53, p16(INK4a), and telomere regulatory functions appear to be differentially regulated during replicative senescence in human and mouse fibroblasts [2]. To which extent premature senescence contributes to the anti-tumorigenic effects of radiation therapy and of various drugs is unknown. If senescence in fact is important, the fact that the mechanism(s) of senescence are not well conserved between mouse and human cells is a concern. Many of the currently used anticancer drugs induce apoptosis of cancer cells, and identification of apoptosis-inducing compounds is of high priority. The control of apoptosis appears to differ between mouse and human cells: BAX knock-out human cells are generally insensitive to anticancer drugs and to radiation [3-5], whereas it appears to be necessary to knock-out both BAX and BAK to achieve the same degree of insensitivity in mouse cells [6]. Why this is so is unclear. What is clear is that mouse fibroblasts grown in monolayer on plastic dishes are not good models for 3-D human tumors proliferating under (often) hypoxic conditions *in vivo*.

## Treatment-sensitive models are widely used in preclinical studies

There are fundamental differences between mouse tumor models and human cancers. Mouse tumors grow very fast and are very angiogenesis dependent. Human tumors do not grow fast and are probably less dependent on angiogenesis. Drugs such as doxorubicin and cisplatin have palliative effects, at best, in patients with recurrent carcinoma but often show very strong activities in xenograft-bearing mice. To make matters worse, treatment-sensitive cell lines are often used in preclinical models. An example is the widespread use of the supersensitive Colo205 cell line in studies of TRAIL.

The use of sensitive models is understandable. It is necessary to demonstrate "proof-of-principle" with regard to treatment strategy. It is remarkable, however, that it is sufficient to present preliminary results on treatment efficiency obtained in highly sensitive models for publication in high impact journals. At the same time, these journals will not publish studies using small clinical materials (< 100 patients), regardless of whether interesting new concepts are presented.

## Irrelevant endpoints are widely used in preclinical studies

Effects of anticancer drugs in pre-clinical models (e.g. xenografts) are often evaluated as retarded growth relative to non-treated control mice. From a clinical perspective, such retarded growth nevertheless represents progressive disease. The commonly accepted clinical end-point is prolonged over-all survival in patients. Although mice have shorter life-spans, it is not difficult to set up relevant end-points also in animal experiments.

## Extreme and irrelevant treatment conditions are widely used in preclinical studies

In many experiments, animals are treated with drugs only days after injection of tumor cells. Such experiments assess drug effects on tumor-take, which is very remote from the clinical situation aiming at tumor regression. Even more remote from clinical realities is the occasional habit of injecting the drug under study into the injection site.

In our hunt for positive results, we often use drug concentrations and radiation doses that are unrealistic. *In vitro *drug concentrations in the high micromolar range are often used. Remarkably, it is often claimed that drugs, even at these concentrations, have single targets. At the same time, most researchers are aware of the problems of unspecific effects using pharmacological inhibitors at more than 5 – 10 μM. It is difficult to accept the concept of a single target when a drug is used at concentrations of 50 – 100 μM. Such concentrations are often used for DNA-damaging drugs. In one study, 500 μM N-methyl-N'-nitro-N-nitrosoguanidine was used to induce alkylating DNA damage, a treatment leading to necrotic cell death [7]. It is very likely that the drug has other targets than DNA at this concentration. The same problems occur in the radiation therapy field. Ionizing radiation clearly induces apoptosis of lymphoid cells. Whether radiation therapy induces acute apoptosis of epithelial cells is, however, controversial [8]. In order to induce apoptosis of carcinoma cells, investigators use fractions of > 10 Gray. We have found reports using doses of 40 Gray in high-ranking journals.

High drug doses are not only used *in vitro*, but also in animal models. Drug doses of 100 mg/kg are not uncommonly used in xenografts models. The highest concentration we found in a rapid survey of recent literature is 1,200 mg/kg. This corresponds to 840 ml intraperitoneal infusion of a 10% solution into a 70 kg patient. Another example is betulinic acid, used at 250 mg/kg to treat mice with melanoma xenografts [9]. Betulinic acid is not in clinical use, and treatment of malignant melanoma is still an unmet medical need. Mice are unable to object to being treated with very high concentrations and volumes of toxic compounds. In mice, toxicity is generally measured as weight loss (typically > 10%) over a limited time period, whereas more sophisticated measures of toxicity are used in humans.

## Aggressive interpretation of data

Many scientists in academia choose to interpret their data quite aggressively. This kind of selective approach may be a successful strategy in hypothesis-building biological research, but does not form a solid foundation for development of clinically useful treatments. There is always a danger that investigators may become devoted to a particular drug, risking to ignore its shortcomings. The development of a drug is a fairly standardized procedure, with extensive ADME (adsorption, distribution, metabolism, and excretion toxicology) studies. Such studies cannot be subjected to aggressive interpretation.

How can the translational process become more effective? Most of us are aware of the problems discussed above, and realize that they impair the process of translational research. One way could be to increase the awareness of journal editors that straightforward papers do not necessarily reflect the complexity of biological systems. As long as "clear results" are presented, high-ranking journals are obviously not always concerned about printing reports where bizarre drug concentrations are used, or where mouse fibroblasts are used as targets for treatment. Since publication in these journals is likely to secure grants for many years, there is an obvious risk that public spending is not used for realistic projects.

Are academic labs suitable for drug development? Both yes and no. Academic laboratories have been successful in providing molecular understanding of sensitivity and resistance necessary for developing new compounds. Academic laboratories have been able to develop anticancer drugs, notably Imatinib for CML. Solid carcinomas are more difficult in terms of more complex targets (i.e. less dependence on one pathway) and of delivery to tumor cells (i.e. ADME). This increased complexity may be difficult to handle for academic groups.

The public expects the cancer research community to cure cancer in humans, and probably care less about cancer in small rodents. It is nevertheless easier to publish papers using knock-out mouse fibroblasts than papers using human tumor cells. We feel that open-access journals such as Radiation Oncology have a large mission to fulfill; to be a role model for publishing relevant data, for open discussion of data and how to use data for making actual progress in translational cancer research. In a longer perspective this will hopefully lead to improvements in the relevance of the data produced.
